# Double deletion of *murA* and *murB* induced temperature sensitivity in *Corynebacterium glutamicum*

**DOI:** 10.1080/21655979.2019.1685058

**Published:** 2019-10-30

**Authors:** Tuo Shi, Qian Ma, Xiaoqian Liu, Yanan Hao, Yanjun Li, Qingyang Xu, Xixian Xie, Ning Chen

**Affiliations:** a Key Laboratory of Industrial Fermentation Microbiology, Tianjin University of Science & Technology, Ministry of Education, Tianjin, P. R. China; b Tianjin Key Laboratory of Industrial Microbiology, Tianjin University of Science & Technology, Tianjin, P. R. China; c College of Biotechnology, Tianjin University of Science & Technology, Tianjin, P. R. China; d National and Local United Engineering Lab of Metabolic Control Fermentation Technology, Tianjin University of Science and Technology, Tianjin, P. R. China

**Keywords:** *Corynebacterium glutamicum*, temperature sensitivity, glutamate, cell wall, metabolomics

## Abstract

Currently, the mechanism of temperature-sensitive production of glutamate in *Corynebacterium glutamicum* has not been clarified. We first found the *murA* and *murB* genes were potentially related to temperature-sensitive secretion of glutamate, which were not existed in a temperature-sensitive mutant. When replenishing *murA* or/and *murB* in the mutant, the temperature sensitivity was weakened. While, their knockout in a wild-type strain resulted in temperature-sensitive secretion of glutamate. Peptidoglycan analysis showed that deletion of *murA* and *murB* decreased the peptidoglycan synthesis. Comparative metabolomics analysis suggested that the variation in cell wall structure resulted in decreased overall cellular metabolism but increased carbon flow to glutamate synthesis, which was a typical metabolism pattern in industrial temperature-sensitive producing strains. This study clarifies the mechanism between *murA* and *murB* deletion and the temperature-sensitive secretion of glutamate in *C. glutamcium*, and provides a reference for the metabolic engineering of cell wall to obtain increased bioproduction of chemicals.

## Graphical Abstract

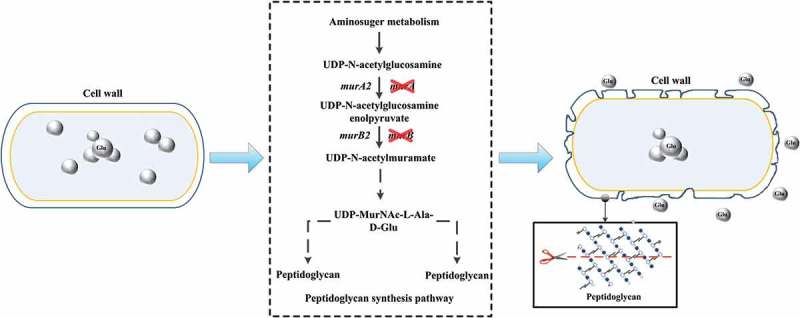

## Introduction

The Gram-positive soil bacterium *Corynebacterium glutamicum* was originally discovered about 60 years ago and is well known as an excellent producer of glutamate [1]. With the development of biotechnology, *C. glutamicum* has been successfully engineered to serve as a versatile workhorse for industrial bioproduction of various chemicals [2–4]. This bacterium has been used to produce more than 4 million tons of diverse amino acids per year, as well as a wide range of other natural and non-natural products, which are used as feed additives, nutritional supplements, pharmaceutical intermediates, biofuels, and polymer building blocks [5].

Wild-type *C. glutamicum* produces little glutamate under normal culture conditions. Using limited biotin supply or adding certain surfactants can induce the secretion of glutamate in industrial fermentation. Without the surfactant addition, the cells grow well but produce little or no glutamate under the condition of excessive biotin supply. The limited biotin supply method cannot be used for fermentation production from biotin-rich raw materials, such as beet molasses [6,7]. There are also many limitations to the method of surfactant addition, because bacteria are extremely sensitive to the addition of surfactants [8]. Addition of penicillin or tetracaine can also induce the secretion of glutamate, but with a relatively low yield. Additionally, drug addition would be cost-prohibitive in fermentation [9,10].

In recent years, the method of temperature-triggered production of glutamate has gradually replaced the traditional biotin-limited-supply method, becoming the preferred method for industrial glutamate production [11]. The temperature-triggered glutamate production process consists of two stages, the cell growth stage at 32–33°C at the early period of fermentation, and the following production stage with temperature shifted to 39°C, when cell growth is inhibited and efficient secretion of glutamate could be observed. Strains used in the temperature-triggered glutamate production method were usually obtained by random mutation and selection, and the mechanism for their high glutamate secretion is still unclear [12–15], which inhibited the further application and improvement of this method.

Studies have shown that the efficient production of glutamate requires the coordination of intracellular metabolism [16,17] and the structural function of the cell membrane and cell wall [18–21] to improve the intracellular synthesis and the secretion processes simultaneously. There have been few studies on the mechanism of glutamate secretion induced by temperature shift. Temperature increase can trigger the up-regulation of a series of intracellular heat shock proteins; affect the processes of carbon source uptake, tricarboxylic acid cycle, and transcriptional regulation [22], and generally inhibit bacterial growth and promote product synthesis. *C. glutamicum* 2262 was reported as a glutamate temperature-sensitive secretion strain [23]. Uy found that temperature increase changed the activity of related enzymes in the glutamate synthesis pathway of this strain [24]. Increase of temperature could not only change the whole intercellular metabolism but could also affect the permeability of cell envelope. Bokas [25] observed different fluidity of the *C. glutamicum* 2262 cell membrane at different temperatures, a positive correlation of the glutamate yield with the fluidity of the cell membrane was revealed, indicating a close relationship between glutamate secretion and the permeability of the cell membrane at increased temperatures.

Besides cell membrane, cell wall also plays an important part in cell permeability, with a variety of important functions, including maintenance of cell shape, protection from mechanical damage, and generation of turgor by restraining the outward osmotic pressure exerted on the cytoplasmic membrane. The secretion of glutamate under shifted temperature fermentation could also be related with the cell wall permeability. Takashi [26] found that a mutation in a single gene of *ltsA* (lysozyme and temperature sensitive) was responsible for its lysozyme sensitivity and temperature sensitivity by complementation tests and DNA sequencing analysis. The knock out of the *ltsA* gene in wild-type *C. glutamiucm* could result in inhibited cell growth and increased glutamate yield when the temperature was increased. The *ltsA* gene product is required for formation or maintenance of the rigid cell wall structure of *C. glutamicum*, the deletion of which could damage the cell wall structure and then facilitate the glutamate secretion. Peptidoglycan is the main component of the cell wall of gram-positive bacteria. Mercier [27] showed that inhibition of the synthesis of peptidoglycan precursor could promote the generation of L-forms (many bacteria, both Gram-positives and Gram-negatives, are capable of switching into a cell wall deficient state, called the ‘L-form’) in both Gram-positive and Gram-negative bacteria.

In this study, *murA* and *murB* genes were targeted as the potential genes affecting the synthesis of peptidoglycan and temperature-sensitive secretion of glutamate by comparative genomics between a temperature-sensitive strain and the wild-type strain. This inference was validated by the knockout of *murA* and *murB* in the wild-type strain, making the wild-type strain obtain temperature sensitive property, with decreased cell growth and increased glutamate secretion under shifted-temperature fermentation. The content analysis of peptidoglycan revealed that the deletion of *murA* and *murB* could reduce the synthesis of peptidoglycan. Comparative metabolomics analysis suggested that the deletion of these two genes lowered the overall cellular metabolism, but the glutamate synthesis process was enhanced. Our study revealed that the temperature sensitive secretion of glutamate was a coordination of cell permeability and the intracellular metabolism.

## Materials and methods

### Strains and culture conditions

The strains and plasmids used in this work are listed in Table 1.

**Table 1.** Strains and plasmids used in this work.

Strains/plasmids	Relevant characteristic	Source
Strains		
ATCC 13,032	Wild type of *Corynebacterium glutamicum*	Lab store
TCCC 11,822 (ST)	Temperature sensitive characteristics of *Corynebacterium glutamicum*	Store in CGMCC 1.16145
AN02	C. *glutamicum* ATCC13,032, Δ*Cgl2610*::Ptuf+*ilvBN*^XV^, Δ*Cgl1890*::Ptuf+*ilvBN*^XV^	Lab store
AN03	AN02, Δ*murA*, Δ*murB*	This study
WT-1	ATCC 13,032, Δ*murA*	This study
WT-2	ATCC 13,032, Δ*murB*	This study
WT-3	ATCC 13,032, Δ*murA*, Δ*murB*	This study
Plasmids		
pXMJ19	Cm^R^, *tac* promoter, cloning vector	Lab store
pK18*mobsacB*	Kan^R^, *lac* promoter, cloning vector	Lab store
pXMurA	Cm^R^, *tac* promoter, *murA* expression plasmid	This study
pXMurB	Cm^R^, *tac* promoter, *murB* expression plasmid	This study
pXMurAB	Cm^R^, *tac* promoter, *murA* and *murB* expression plasmid	This study

Slant culture medium [28,29] contained 10 g/L peptone, 5 g/L yeast extract, 10 g/L beef paste, 2.5 g/L NaCl, 0.5 g/L MgSO_4_, 1 g/L KH_2_PO_4_, and 20 g/L agar. The cultures were grown at 32°C for over 12 h. Then, the seed was cultured in seed medium containing 40 g/L glucose, 20 mL/L corn steep liquor, 2 g/L KH_2_PO_4_, 2 g/L MgSO_4_, 2.5 mg/L MnSO_4_, 2.5 mg/L FeSO_4_, 0.2 mg/L V_B1_, and 0.5 mg/L V_H_ at 32°C with shaking at 200 r/min for 6–8 h. Following which, the fermentation process was performed in 500 mL shake flasks containing 50 g/L glucose, 20 mL/L corn steep liquor, 4.5 g/L KH_2_PO_4_, 2 g/L MgSO_4_, 30 mg/L MnSO_4_, 30 mg/L FeSO_4_, 0.3 mg/L V_B1_, and 0.3 mg/L V_H_ for 28–32 h.

### Construction of plasmids and recombinant strains

Plasmids were assembled and genes were knocked out as described [30]. The recombinant strains WT-1, WT-2 and WT-3 were obtained by knocking out *murA, murB* and both *murA* and *murB*. All primers were synthesized by Genewiz (Beijing, China) and are listed in Supplementary Table 1. Gene sequencing was carried out by Novogene (Tianjin, China).

### Fermentation experiment

In order to verify whether the deletion of the two genes can confer temperature sensitivity and promote the efflux of glutamate, we performed flask fermentation experiments of overexpression strains and recombinant strains. These strains were separately inoculated in 500 mL flask. Cell growth was monitored by measuring the optical density at 600 nm (OD_600_). The initial fermentation temperature was 32°C, and when the ΔOD_600_ reached 15 to 18, the temperature was shifted to 39°C. The fermentation temperature of the control group remained constant at 32°C. The pH was automatically controlled at 7.0–7.2 by the addition of liquid ammonia. The fermentation period was 28–32 h. When glucose in the fermentation medium was consumed, 60% glucose solution was added at an appropriate rate to maintain a glucose concentration below 5 g/L.

### Analytical methods

Glucose concentration was measured using an SBA-40E biological sensor (Shandong Academy of Sciences, Shandong, China). The glutamate concentration in shake-flask was quantified by isocratic HPLC (Thermo Scientific UltiMate 3000) using a Gemini C_18_ column (Phenomenex, USA). An acetonitrile/water mixture (2:98 v/v) was used as the mobile phase at a flow rate of 1 mL/min. The wavelength of the UV detector and the temperature of column oven were 270 nm and 30°C, respectively.

### Observation of cell morphology

The cell morphology of ST and ATCC 13032 was observed by scanning electron microscopy. The cells were treated and observed according to the following procedures: (1) The ST and ATCC 13032 cultures were centrifuged at 4000 r/min for 2 min; (2) The precipitation was cleaned by deionized water and then centrifuged at 4000 r/min for 5 min. (3) Repeat step ‘(2)’ for 2–3 times. The precipitation was resuspended in 1 mL 2.5% (v/v) glutaraldehyde, and was placed at 4°C for 3 h; (4) The sample was again subjected to centrifugation and the precipitated material was then washed twice with deionized water and centrifuged at 4000 r/min for 5 min; (5) The precipitation was sequentially eluted with 30%, 50%, 70%, 90%, and anhydrous ethanol for 10 min; (6) An appropriate amount of the above mixture was then transferred to a slide glass and allowed to dry naturally. (7) The slide was pasted on the metal sample platform using conductive adhesive and then vacuum coating. The cells were observed under scanning electron microscope.

### Extraction and determination of peptidoglycan

As *murA* and *murB* genes are crucial in the synthesis pathway of peptidoglycan, the deletion of these two genes is likely to result in incomplete synthesis of peptidoglycan in *C. glutamicum* ATCC 13032. To verify whether the lack of these genes can lead to the inhibition of peptidoglycan synthesis, we extracted and measured peptidoglycan in WT-1, WT-2, WT-3 and *C. glutamicum* ATCC 13032. The extraction and measurement were performed as follows [31,32]: (1) The fermentation broth was centrifuged to obtain 20 g of precipitated material. This material was then suspended in 200 mL of 10 g/L trichloroacetic acid (TCA) solution. (2) The samples were incubated in a water bath at 75°C for 25 min and remove the phosphorus acid from the cell wall. (3) The samples were then cooled to room temperature, and then centrifuged at 8000 r/min for 10 min to collect the cell material without teichoic acid. (4) The precipitated material was then washed with distilled water until reaching neutral pH. The precipitate was added to the trypsin phosphate buffer (3 mg/mL trypsin in PBS, pH = 8.0) at a solid to liquid ratio of 1:5 (quality volume ratio). The solution was then cultured with agitation at 120 r/min and 37°C for 12 h. (5) The insoluble material was removed by centrifuging at 1500 r/min for 5 min. The supernatant was again centrifuged and then protein-free cell precipitation was obtained. The precipitate was washed three times with distilled water, and then mixed with a chloroform-methanol mixture (chloroform: methanol = 1:2) for 6 h to remove lipid substances. (6) The mixed solution was centrifuged, the precipitation was dehydrated by absolute alcohol and allowed to dry naturally, and then the light brown peptidoglycan extract was obtained.

Peptidoglycan yield (%) = (extracted peptidoglycan quality/pre-extraction dry cell weight) X 100

### Metabolomics analysis method

Cells were harvested by centrifugation and washed with PBS buffer and ddH_2_O for metabolomics analysis. Cell debris was obtained by grinding with mortar and pestle in liquid nitrogen. Fifty-milligram cells were suspended in 1 mL extraction liquid (methanol/water, 1:1, v/v) for extraction. One hundred microliter of the supernatant of the extraction was mixed with 20 μL internal standard (succinic d_4_ acid, 0.1 mg/mL). The mixture was lyophilized and subsequent two-stage chemical derivatization was performed.

The detection of metabolites was carried out using Masslynx software (Version 4.1, Waters Corp., USA). Metabolites identification was carried out by searching the National Institute of Standards and Technology mass spectral library (NIST 2010). The quantification of metabolites was conducted by calculating the areas under respective peaks after normalization against the internal standard. Hierarchy clustering analysis was performed by MetaboAnalyst 4.0 (https://www.metaboanalyst.ca/MetaboAnalyst/faces/home.xhtml).

## Results

### *Genomic comparison of* C. glutamicum *ST and* C. glutamicum *ATCC13032*

**Figure 1.**
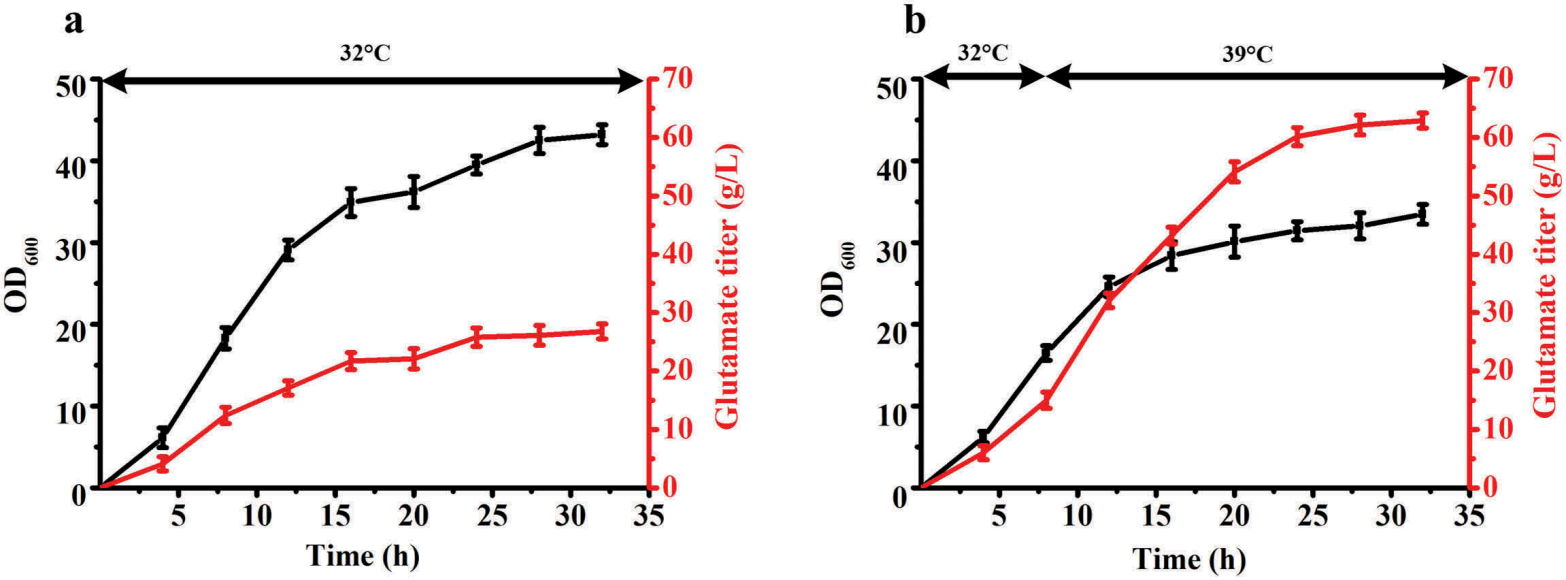
The fermentation results of *C. glutamicum* ST. (**a)** The growth curve and glutamate titer curve of 32°C fermentation. (**b)** The growth curve and glutamate titer curve of 32°C to 39°C fermentation. The fermentation temperature was beginning at 32°C, then increased the temperature to 39°C when OD_600_ reached 16–18. Three sets of parallel repeated experiments were carried out at two different temperatures.

Glutamate is industrially produced worldwide, making it the amino acid with the highest annual production [33]. The main production is fermentation of a temperature-sensitive mutant *C. glutamicum*. The cells of the temperature-sensitive mutant strain convert from growth to acid production type in response to a temperature increase, resulting in high accumulation of glutamate [26,27]. Compared to the traditional fermentation of glutamate, this is a more efficient fermentation process with higher glutamate titer and yield. However, the mechanism by which temperature-sensitive secretion of glutamate is still not clear. In the previous study, our lab applied mutagenesis and selected a high-yield glutamate-producing *C. glutamicum* ST with temperature-sensitive characteristics. The shake flask fermentation results of ST are presented in Figure 1. The growth rate of ST decreased when the fermentation temperature increased from 32°C to 39°C when the culture reached a cell density of OD_600_ = 15–18. Glutamate began to accumulate in abundance with an increase of the temperature from 32°C to 39°C. Temperature sensitivity is a special property that apparently can regulate cell growth and metabolism and promote the synthesis of products under the condition of increasing temperature [34].

**Figure 2.**
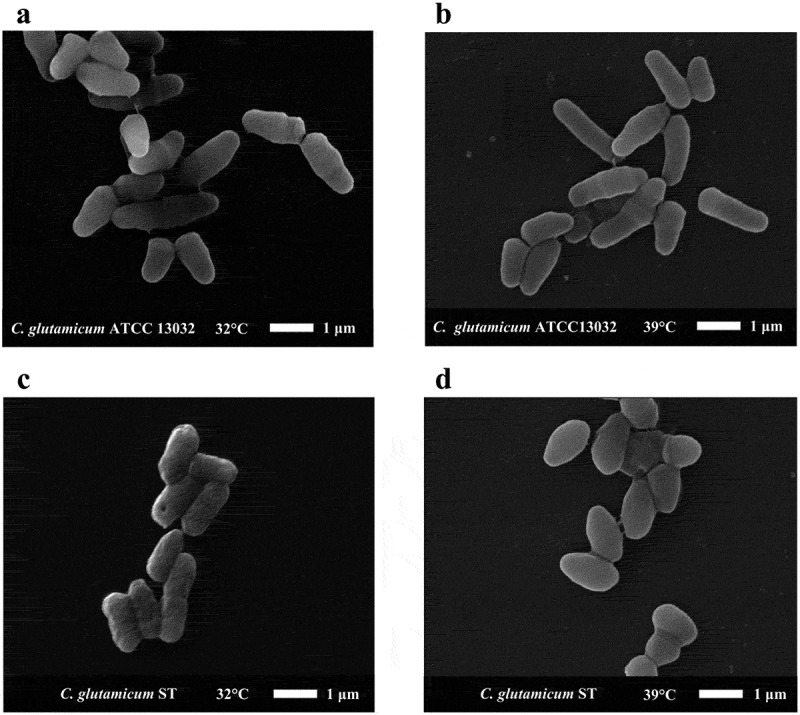
The cell morphology of *C. glutamicum* ST and *C. glutamicum* ATCC 13032 under electron microscope. We selected representative cell morphology at different temperatures for observation. (**a)** The morphology of *C. glutamicum* ATCC 13032 at 32°C. (**b)** The morphology of *C. glutamicum* ATCC 13032 at 39°C. (**c)** The morphology of *C. glutamicum* ST at 32°C. (**d)** The morphology of *C. glutamicum* ST at 39°C.

We observed the cell morphology of ST and wild type (ATCC13032) at different temperatures by scanning electron microscope. As shown in Figure 2(a), the cell morphology of ATCC 13032 at 32°C is typical for *C. glutamicum*. The cell length was about 1 μm-2 μm, and cell width was about 0.6 μm. And the cell length was almost unchanged at 39°C (Figure 2(b)). The cell surface was smooth at 32°C and 39°C (Figure 2(a,b)). However, the ST cell surface was not smooth at 32°C. The cell length was less than that of ATCC 13032, about 1 μm −1.5 μm, and the cell width was about 0.5 μm (Figure 2(c)). Compared to the cell morphology of ST at 32°C, the cells became shorter and more round at 39°C. The cell length was about 0.8 μm, the cell width was about 0.7 μm, and the cell surface became smooth (Figure 2(d)).

In order to elucidate the temperature-sensitive mechanism, we carried out whole genome sequencing of ST. And there was a significant change in cell morphology in ST at different temperatures, so we focused on comparing genes in ST and ATCC13032 whose functions are related to cell wall and cell membrane.

Comparative genomics was performed by blastp software, and 93 differential genes were involved in cell wall and cell membrane-related synthesis (Supplementary Table 2). Of these 93 genes, only *murA* and *murB* genes were deleted in ST, which could affect the synthesis of peptidoglycan (Supplementary Figure 1). However, there is another isoenzyme for these two deleted genes, respectively. So, the deletion of these two genes cannot completely block the synthesis of cell wall, explaining the viability of the strain (Supplementary Figure 1). However, the deletion of these genes may decrease the level of peptidoglycan synthesis during strain growth, resulting in incomplete cell wall synthesis, alteration of cell surface structure, or the formation of an incomplete cell wall structure. As a result of this alteration, natural channels of glutamate transport protein could form. The natural channels of glutamate transport protein promote glutamate secretion to the extracellular environment, which can reduce the intracellular concentration of glutamate and promote the accumulation of secreted glutamate.

### *Effect of overexpression of* murA *or/and* murB *on growth and glutamate production of ST*

**Figure 3.**
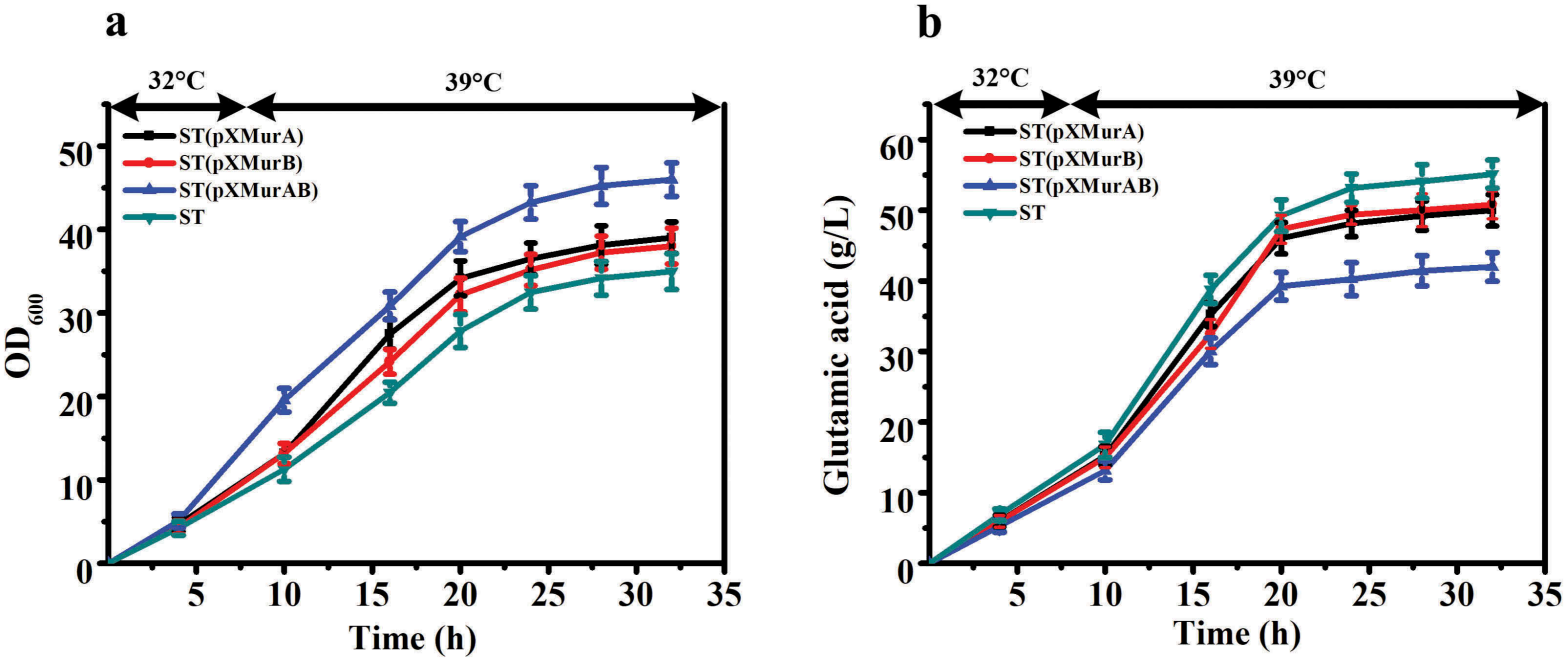
Effect of overexpression of *murA* or/and *murB* on growth and glutamate production of ST. **(a)** Cell growth of these strains. **(b)** Glutamate production of these strains.

In order to confirm that *murA* and *murB* are related to the temperature sensitive characteristics of this strain, further experiments were carried out on the two genes. We separately overexpressed *murA* or *murB* gene or co-overexpressed them in ST to explore whether increased amounts of the gene products would affect the temperature sensitive characteristics and glutamate producing ability of this strain. The fermentation results are illustrated in Figure 3. The OD_600_ of ST, ST (pXMurA), ST (pXMurB), and ST (pXMurAB) reached the maximum value at 32 h. The OD_600_ of ST (pXMurAB) strain was increased by 23.9% compared with that of ST (Figure 3(a)). At the end of fermentation, the glutamate production of the control strain ST was 55.1 g/L, production of ST (pXMurA) was 49.9 g/L, ST (pXMurB) produced 50.7 g/L, and ST (pXMurAB) produced only 41.9 g/L. The ST (pXMurAB) strain had the lowest production of glutamate, a decrease of 23.8% compared to the standard strain (Figure 3(b)). To summarize, we found that the growth and glutamate production of strains was inversely affected by the presence of the *murA* or *murB* overexpression cassette. The growth and glutamate production were more affected in the strain that contained both the *murA* and *murB* overexpression cassettes. Specifically, the presence of *murA* and *murB* removed the temperature sensitive characteristics to a certain extent with increased cell growth and decreased glutamate production. Thus, we speculated that the functions of *murA* and *murB* might be related to the temperature-sensitive characteristics of *C. glutamicum* ST.

### *Effect of deletion of* murA *or/and* murB *on growth and amino acid production*

**Figure 4.**
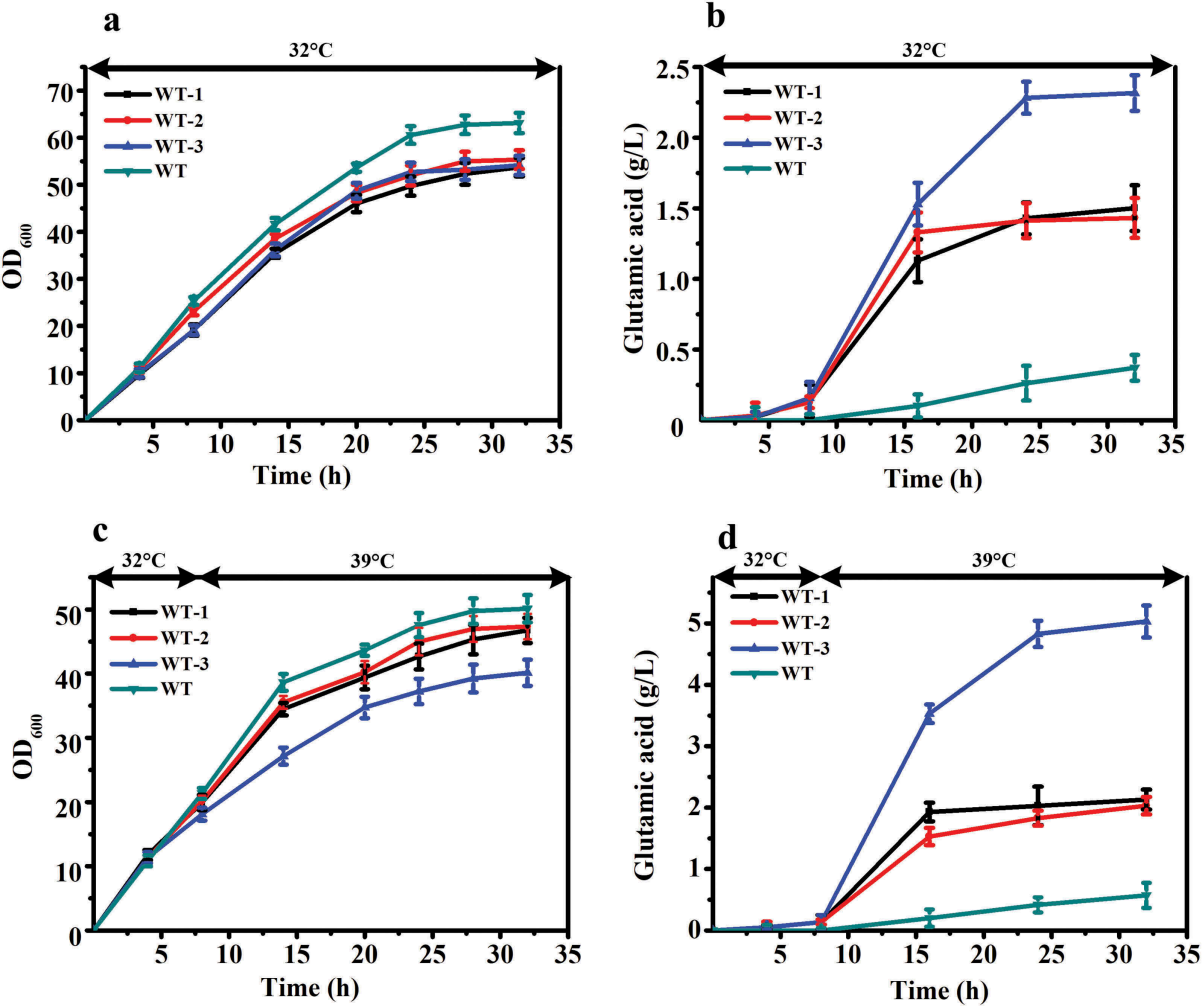
Comparison of fermentation properties among WT (*C. glutamicum* ATCC 13032) and its *murA* or/and *murB* deletion strains (WT-1 (WT, Δ*murA*), WT-2 (WT, Δ*murB*), WT-3 (WT, Δ*murAB*)) under different fermentation temperature. (**a)** Cell growth of these strains at 32°C. **(b)** Glutamate production of these strains at 32°C. (**c)** Cell growth of these strains first at 32°C, and shifted to 39°C when the ΔOD_600_ reached 15 to 18. (**d)** Glutamate production of these strains first at 32°C, and shifted to 39°C when the ΔOD_600_ reached 15 to 18.

To further verify the relationship between these two genes and the temperature sensitive characteristics, we knocked out these two genes from wild-type *C. glutamicum* ATCC 13032. The genes of *murA* or/and *murB* were knocked out individually and together, resulting in strains WT-1, WT-2, and WT-3, respectively. The results of shake flask fermentation showed that WT-1, WT-2, WT-3, and the wild-type ATCC 13032 exhibited comparable cell growth and glutamate titer at 32°C and 32 – 39°C (Figure 4).

The OD_600_ of ATCC 13032 was higher than that of the other three strains at the end of fermentation at 32°C (Figure 4(a)). However, its production of glutamate was the lowest, only 0.4 g/L. The glutamate titer of WT-1, WT-2, and WT-3 reached 1.5 g/L, 1.4 g/L and 2.3 g/L, increases of 305.4%, 278.4%, and 521.6%, respectively, relative to the wild-type strain (Figure 4(b)).

The cells of ATCC 13032 also grew fastest than the other three strains after the temperature increased. The OD_600_ of all four strains reached maximum value at 32 h. The lowest value was 46.7 for WT-3, which was decreased 19.9% compared with ATCC 13,032. WT-1 and WT-2 were also decreased, 6.7% and 5.5%, respectively (Figure 4(c)). The glutamate-production rate of WT-3 increased rapidly after temperature increased, and reached maximum value of 5.1 g/L at 32 h. ATCC 13032, WT-1, and WT-2 reached maximum value of 0.6 g/L, 2.1 g/L, and 2.0 g/L, respectively (Figure 4(d)). There was a significant difference in production between the knocked out strains and wild-type ATCC 13032. The glutamate yield of WT-3 increased by 782.5% compared with that of ATCC 13032. The glutamate titers were also increased in WT-1 and WT-2, by 273.7% and 256.1%, respectively, compared to that of ATCC 13032.

Interestingly, compared with fermentation at 32°C, when the fermentation temperature was 32°C-39°C, the OD_600_ of all strains decreased, and the glutamate titer increased. For the WT-3 strain, the OD_600_ decreased by 25.9%, and the glutamate titer increased by 121.7%. The results above indicated that removal of *murA* or/and *murB* decreased OD_600_ and increased glutamate titer at 32°C fermentation temperature. And, when temperature was increased during the fermentation process, this phenomenon was more obvious.

**Figure 5.**
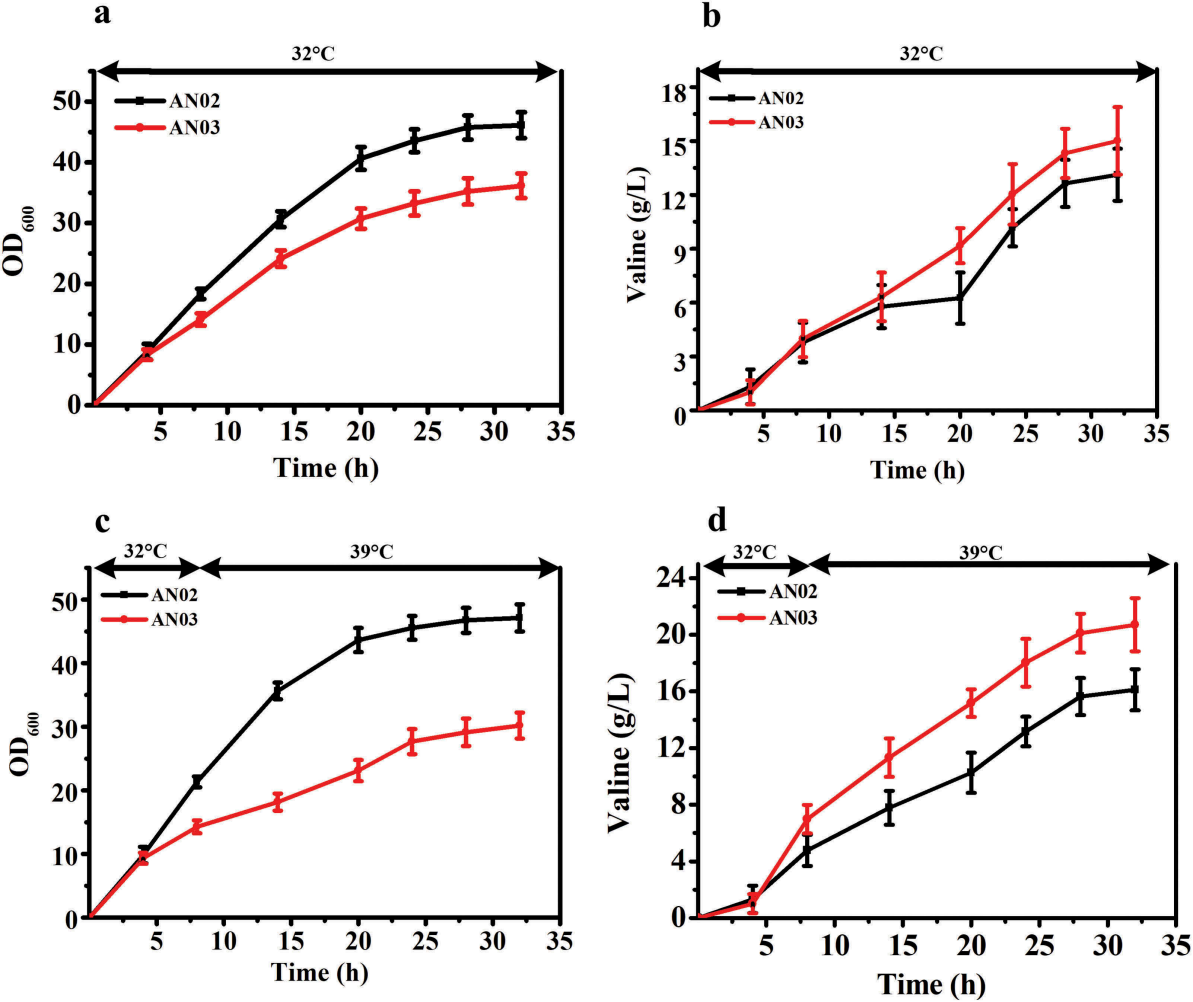
Effects of deletion of *murAB* genes in a valine-producing strain AN02. (**a)** Comparison of cell growth between AN02 and its *murAB* deletion strain AN03 at 32°C. (**b)** Glutamate production of two strains at 32°C. (**c)** Cell growth of two strains first at 32°C, and shifted to 39°C when the ΔOD_600_ reached 15 to 18. (**d)** Glutamate production of two strains first at 32°C, and shifted to 39°C when the ΔOD_600_ reached 15 to 18.

We used the same method to knock out both the *murA* and *murB* genes in a valine-producing strain AN02, and obtained the strain AN03. The shaking flask results showed the same trend as the above fermentation experiments (Figure 5). The OD_600_ of AN03 and AN02 reached maximum value after 32 h of fermentation at 32°C, and the OD_600_ of AN03 was decreased by 6.9% compared with that of AN02 (Figure 5(a)). However, the valine production of AN03 was increased by 14.8% compared with that of AN02 (Figure 5(b)). Similarly, when the temperature in the fermentation process was increased, AN03 exhibited a decrease of 12.5% in OD_600_ compared with AN02, and valine production increased by 58.3% (Figure 5(c,d)). This result shows that like the effect of the knockout of *murA* and *murB* genes to make *C. glutamicum* ATCC 13032 temperature sensitive and improve its yield of glutamate, this method can also be used to improve the yield of other amino acids such as valine.

### *Effects of* murA *or/and* murB *deletion on peptidoglycan synthesis*

**Figure 6.**
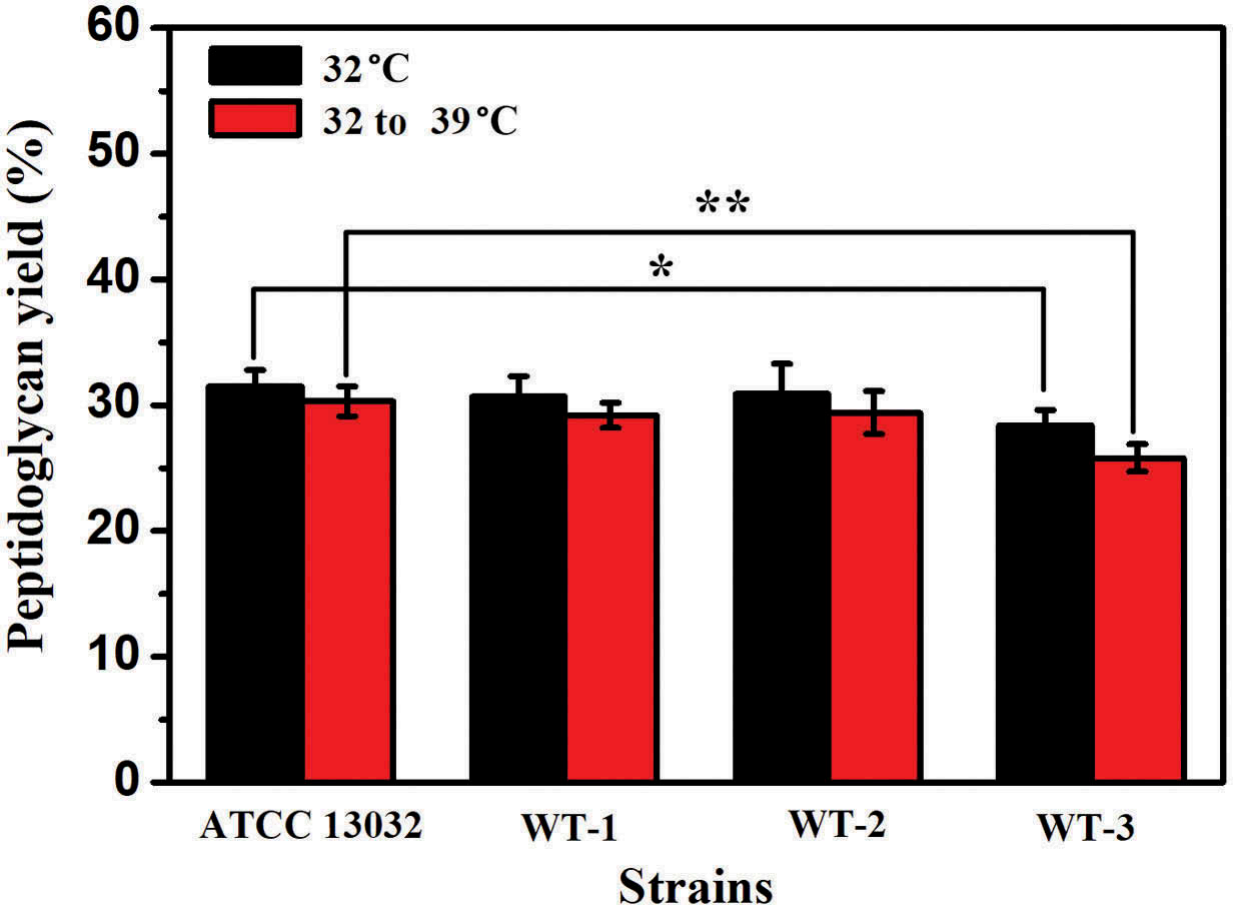
The results of peptidoglycan content in ATCC 13032 and recombinant strains. Peptidoglycan yield (%) = (extracted peptidoglycan quality/pre-extraction dry cell weight) X 100. The significant difference between the data from each recombinant strain and ATCC 13032 was analyzed. * indicates *p* < 0.05, ** indicates *p*< 0.01.

We speculated that the deletion of *murA* or/and *murB* affected the synthesis of peptidoglycan, affecting the integrity of cell wall, which plays a role in maintaining cell morphology. To further verify the correlation between the deleted genes and temperature sensitive characteristics, especially the effects on peptidoglycan synthesis, the cell wall peptidoglycan was extracted and its content was determined. As shown in Figure 6, compared with that of ATCC13032, the peptidoglycan content of WT-1, WT-2, and WT-3 were decreased by 2.5%, 2.0%, and 9.8% at 32°C, respectively. The peptidoglycan levels of WT-1, WT-2, and WT-3 were decreased by 3.6%, 3.0% and 14.9% compared with that of ATCC 13032 at shifting temperature, from 32°C to 39°C. Compared with the 32°C fermentation, when the fermentation temperature was 32–39°C, the content of peptidoglycan in all strains decreased, but this decrease was more obvious in the recombinant strains, especially WT-3, which exhibited a decrease in the content of peptidoglycan of 9.2%. These results showed that the knockout of *murA* or/and *murB* did inhibit the synthesis of peptidoglycan, and the deleterious effects of insufficient peptidoglycan were more obvious at increased temperature.

### *Comparative metabolomics analysis of WT and the* murAB *deletion strain (WT-3)*

**Figure 7.**
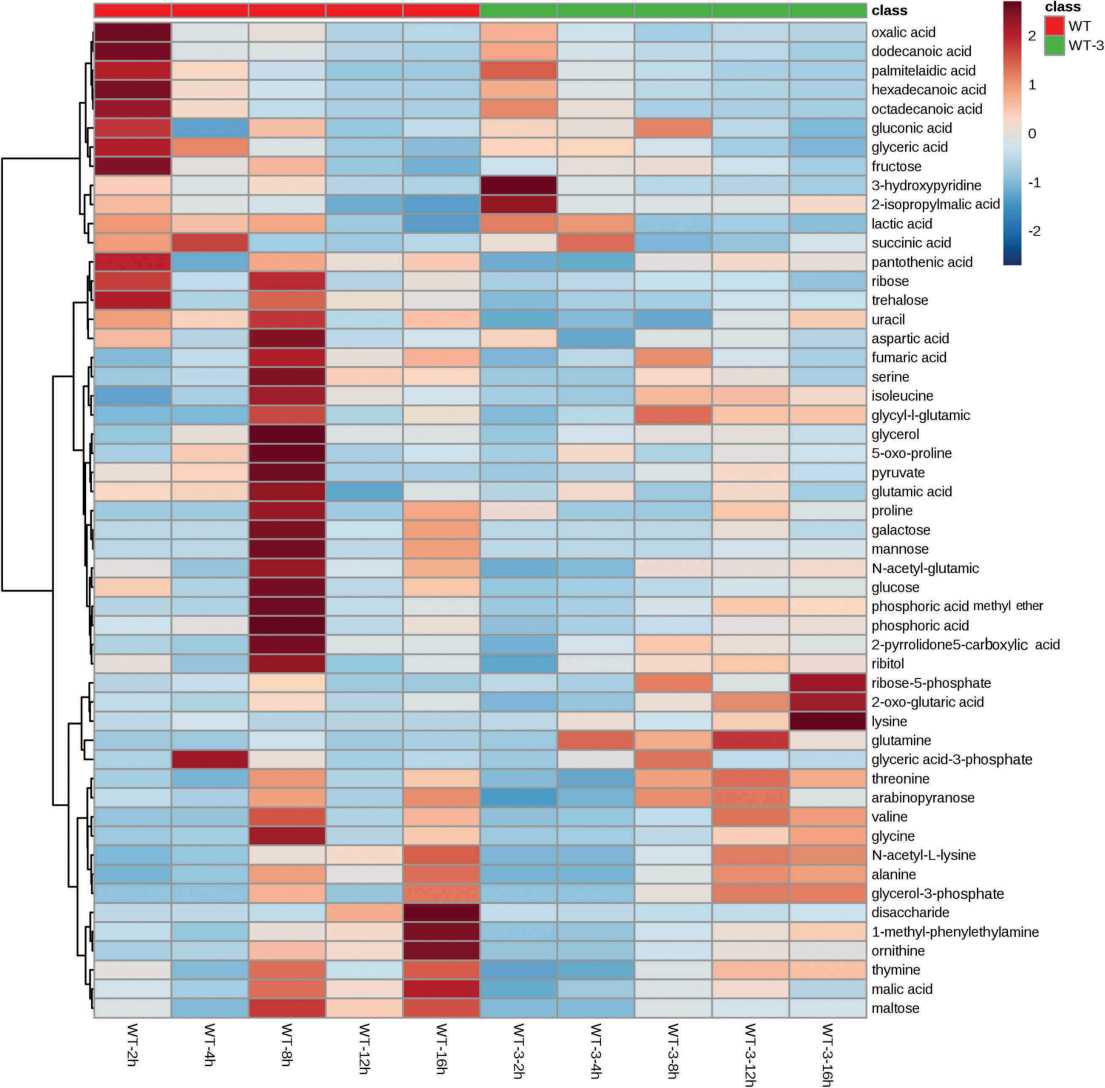
Hierarchy clustering analysis of metabolites in WT and WT-3.

In order to clarify the metabolomics variation resulting from the deletion of *murAB* in the wild type strain, comparative metabolomics analysis of WT and WT-3 at different fermentation time was performed. In total, 52 metabolites were identified. To give an overall comparison of the metabolomics data, hierarchy clustering analysis (HCA) was performed, and the result is shown in Figure 7. From the clustering result, it was clear that different groups of variation existed. The majority of the metabolites in WT-3 had lower level compared with those in WT, consistent with the poor cell growth of WT-3 at temperature-shifting fermentation. This group of metabolites includes metabolites related with the latter part of TCA cycle (succinic acid, fumaric acid), amino acids derived from glutamate (proline, 5-oxo-proline), and aspartic acid family amino acids (aspartic acid, isoleucine). While, in the second group, 2-oxo-glutaric acid, the direct precursor of glutamate, showed significant increase in WT-3, suggesting more carbon flowing to the synthesis of glutamate, instead of flowing to the latter part of TCA cycle. Because of the increased cell permeability of WT-3, the intracellular glutamate could be efficiently exported, as a result of which, the intracellular glutamate level could maintain a low level and thus increased the driving force for the synthesis of glutamate. These results could explain the temperature sensitive property of WT-3, showing poor cell growth and enhanced glutamate secretion.

## Discussion

### Increased temperature is favored in the industrial production

In recent years, the production process of glutamate secretion induced by increased temperature has gradually replaced the method of fermentation with added biotin or other processes to become the production method used for most industrial production of glutamate [35]. Compared with the methods of limited biotin and surfactant addition, this method has the following obvious advantages: high conversion rate, simple operation, inexpensive, lower possibility of contamination by miscellaneous bacteria, and easy industrial application [36–38]. In addition to glutamate, the temperature increase method has also been applied in the fermentation of xylanase, jinggangmycin, lovastatin, and other products [39]. We knocked out *murA* and *murB* genes in a valine-producing strain and found that the resulting strain obtained temperature-sensitive characteristics with improved production of valine. Studies on the cell wall peptidoglycan and cell membrane of *C. glutamicum* will contribute to a better understanding of temperature-sensitive characteristics and the mechanism of acid amino secretion [40].

### Incomplete synthesis of peptidoglycan facilitates the temperature sensitive secretion of glutamate

In this study, we identified the importance of *murA* and *murB* as key genes in the biosynthesis pathway of cell wall peptidoglycan (Supplementary Figure 1) by comparing and analyzing the genomes of temperature-sensitive *C. glutamicum* ST and *C. glutamicum* ATCC 13032. The lack of function of these two genes contribute to the temperature-sensitive characteristics of *C. glutamicum*, as the deletion of *murA* and *murB* makes *C. glutamiucm* ATCC 13032 obtain temperature-sensitive characteristics and greatly enhances the secretion of glutamate.

There have been few studies of the relationship between cell wall or cell membrane and temperature-sensitive glutamate secretion. Radmacher [41] studied the mechanism by which addition of ethanolamine promotes glutamate secretion of *C. glutamicum* ATCC 13032 and found that ethanolamine inhibited the synthesis of the mycolic acid layer on the cell wall. These changes in the cell wall structure caused corresponding changes in the cell membrane, and the large amount of glutamic acid secretion is closely related to the structure and function of cell membrane and cell wall. Du [42] found that gram-positive bacteria contain two *murA* genes and both genes encode active enzymes that can substitute for each other, but that the presence of *murA* function is essential to the organism. This finding is consistent with the results of this study. We found isoenzymes of *murA* and *murB* through genomic comparison, which called *murA2* and *murB2*, and only *murA* and *murB* were missing in *C. glutamicum* ST. We speculated that with knockout of either the *murA* and *murB* genes in *C. glutamicum* ATCC 13032, the remaining isoenzyme could still catalyze the synthesis of peptidoglycan normally, but the catalytic efficiency would decrease. However, when the temperature increased, the activity of the remaining isoenzymes would be affected, leading to a more dramatic phenotype. Under these conditions, the synthesis of peptidoglycan would be greatly affected, which has been proven by the peptidoglycan content decrease.

### Intracellular metabolism, cell permeability and the secretion of glutamate should be coordinated in temperature-sensitive production of glutamate

Comparative metabolomics analysis revealed that the deletion of *murA* and *murB* genes under-shifted temperature fermentation greatly affected the intracellular metabolism of the mutant strain. The overall metabolism of the *murA* and *murB* deletion mutant showed a declined tendency, which was coherent with its inhibited cell growth. But the synthesis of precursor of glutamate was enhanced, suggesting that with the efficient export of glutamate, the intracellular glutamate pressure was lowered, and could act as a driving force for the upstream synthesis of glutamate. The results of this study showed that the intracellular metabolism, cell envelope structure and the secretion of glutamate should be coordinated to obtain the temperature-sensitive production of glutamate. This is an important principle that should be considered in future metabolic engineering of industrial glutamate strains.

## Conclusion

In this study, overexpression of *murA* and *murB* genes in *C. glutamiucm* TCCC 11822 and deletion of *C. glutamiucm* ATCC 13032 were used to determine the relationship between *murA* and *murB* genes and temperature-sensitive characteristics of *C. glutamiucm* TCCC 11822. The results of scanning electron microscopy and gene analysis showed that *murA* and *murB* genes were the first two genes in the pathway of peptidoglycan synthesis. Their deletion would inhibit the synthesis of peptidoglycan, thus affecting cell wall synthesis and changing cell permeability. Fermentation experiments and metabolomics studies have confirmed that the deletion of *murA* and *murB* gene can inhibit the growth of bacteria, increase cell permeability, and effectively export intracellular glutamate, so that the intracellular glutamate level can be maintained at a low level, thereby increasing the driving force of glutamate synthesis and promoting glutamate secretion.

## Research Highlights

- Deletion of *murAB* induced temperature sensitivity of the wild type.
- Replenishing of *murAB* weakened the temperature sensitivity of the mutant.
- Cell wall structure variation affected intracellular metabolism.
