# Supplementary material for: Double deletion of murA and murB induced temperature sensitivity in Corynebacterium glutamicum
Source: Bioengineered. 2019 Oct 30;10(1):561–73. doi: 10.1080/21655979.2019.1685058 (PMC6844371; doi:10.1080/21655979.2019.1685058)
Supplement: Supplemental Material [file kbie-10-01-1685058-s001.zip › Supplementary Table 1 Primers of knocking out murA and murB.docx]

Supplementary Table 1 Primers of knocking out *murA* and *murB*

| Name | Primers | Restriction site |
| --- | --- | --- |
|  |  |  |
| *murA*-1 | CCAAGCTTGGTTGGTCCTGATTGGGTAGAAGC | *Hind*III |
| *murA*-2 | TAAATCCGCACGATACTACCCCTCAACGCC |  |
| *murA*-3 | TAGTATCGTGCGGATTTAACTATCAGAGAAGG |  |
| *murA*-4 | CGGAATTCCGCTCACCGATTCCACGACGC | *EcoR*I |
| *murB*-1 | CCAAGCTTGGGATTGGCTTCTGCTTTCCGT | *Hind*III |
| *murB*-2 | CTAAAGTGTCCTCATGGCAACGCGTAC |  |
| *murB*-3 | CATGAGGACACTTTAGAATCCCTCGACGGT |  |
| *murB*-4 | CGGAATTCCGGCGGCCTTCGGTGTATTTT | *EcoR*I |
| *murAB*-1 | AAGCTTTGCATGGGCAGGGACTCT | *Hind*III |
| *murAB*-2 | ATTCGGCCGCCGCGAGAAGTCTAGTAGC |  |
| *murAB*-3 | CTCGCGGCGGCCGAATTGGAAACGAT |  |
| *murAB*-4 | GAATTCGTGAAGCTGTTCCATCTGTCG | *EcoR*I |
