# Supplementary material for: Double deletion of murA and murB induced temperature sensitivity in Corynebacterium glutamicum
Source: Bioengineered. 2019 Oct 30;10(1):561–73. doi: 10.1080/21655979.2019.1685058 (PMC6844371; doi:10.1080/21655979.2019.1685058)
Supplement: Supplemental Material [file kbie-10-01-1685058-s001.zip › Supplementary Fig caption.docx]

Supplementary Fig. 1 The synthesis pathway of peptidoglycan in *C. glutamicum* cell wall. The genes with red and framed in red represent the deletion genes in ST. The *murA* and *murB* genes were first two key genes in peptidoglycan synthesis. The *murA2* and *murB2* genes were isozymes of *murA* and *murB*.
