# Supplementary material for: Double deletion of murA and murB induced temperature sensitivity in Corynebacterium glutamicum
Source: Bioengineered. 2019 Oct 30;10(1):561–73. doi: 10.1080/21655979.2019.1685058 (PMC6844371; doi:10.1080/21655979.2019.1685058)
Supplement: Supplemental Material [file kbie-10-01-1685058-s001.zip › Supplementary Table 2 The results of genomic comparison.docx]

Supplementary Table 2 The results of deletion genes and mutant genes in cell wall by compared between ST and ATCC 13032

|  | WT | ST | Function |
| --- | --- | --- | --- |
| ***murA*** | **+** | - | UDP-N-acetylglucosamine enolpyruvyl transferase |
| ***murB*** | **+** | - | UDP-N-acetylmuramate dehydrogenase |
| ***dac*** | **+** | ***** | D-alanyl-D-alanine carboxypeptidase |
| ***murA2*** | **+** | ***** | UDP-N-acetylglucosamine 1- carboxyvinyltransferase |
| ***murC*** | **+** | ***** | UDP-N-acetylmuramate--L-alanine ligase |
| ***murD*** | **+** | ***** | UDP-N-acetylmuramoyl-L-alanyl-D-glutamate synthetase |
| ***murE*** | **+** | ***** | UDP-N-acetylmuramoylalanyl-D-glutamate--2,6-diaminopimelate ligase |
| ***murG*** | **+** | ***** | undecaprenyldiphospho-muramoylpentapeptide beta-N-acetylglucosaminyltransferase |
| ***mraY*** | **+** | ***** | phospho-N-acetylmuramoyl-pentapeptide-transferase |

-：deletion；*：mutation
