# Supplementary figures and images for: Double deletion of murA and murB induced temperature sensitivity in Corynebacterium glutamicum
Source: Bioengineered. 2019 Oct 30;10(1):561–73. doi: 10.1080/21655979.2019.1685058 (PMC6844371; doi:10.1080/21655979.2019.1685058)

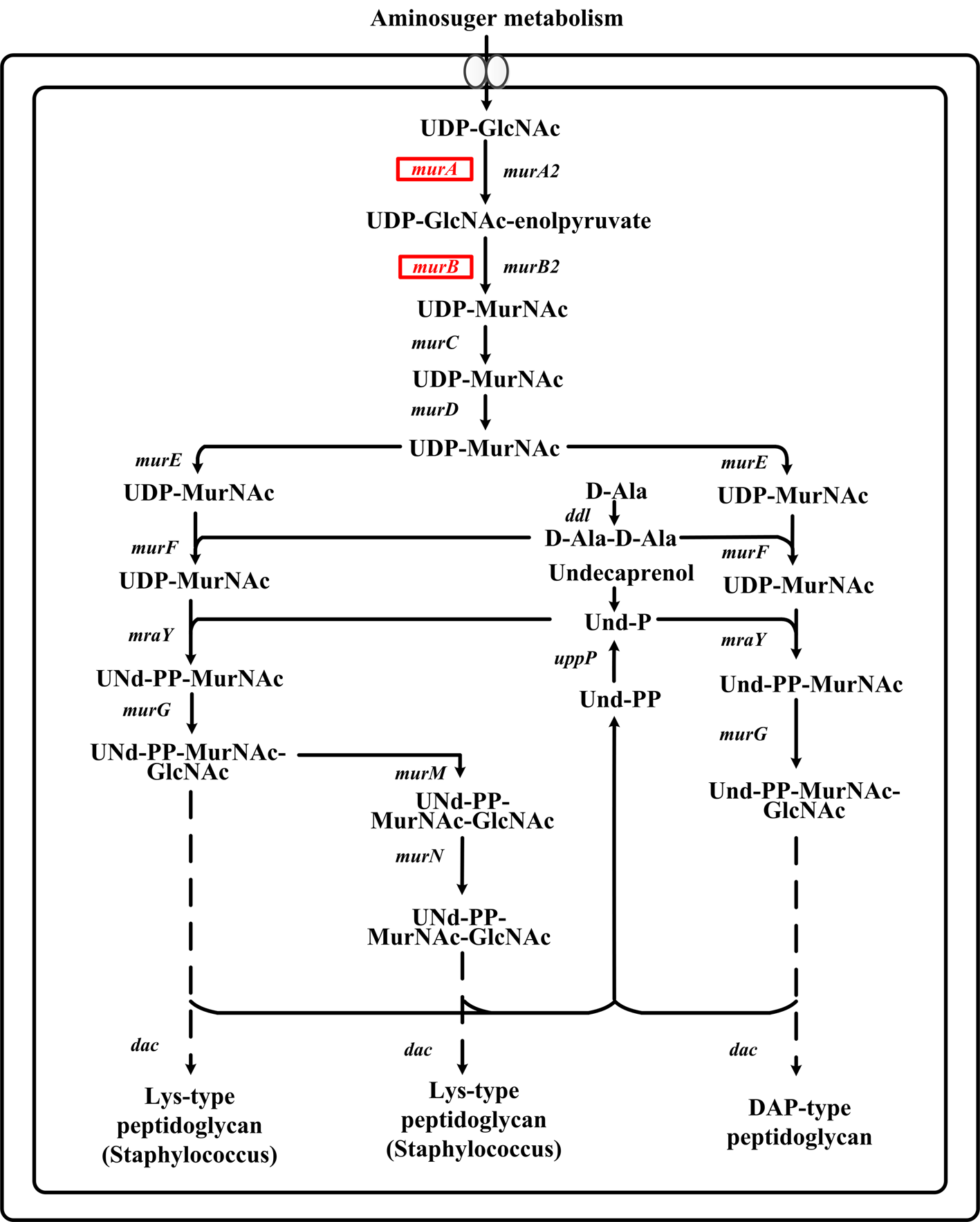

Supplement: Supplemental Material [file kbie-10-01-1685058-s001.zip › Supplementary Figure 1.tif]
